# A Machine Learning and Blockchain Based Efficient Fraud Detection Mechanism

**DOI:** 10.3390/s22197162

**Published:** 2022-09-21

**Authors:** Tehreem Ashfaq, Rabiya Khalid, Adamu Sani Yahaya, Sheraz Aslam, Ahmad Taher Azar, Safa Alsafari, Ibrahim A. Hameed

**Affiliations:** 1 Department of Computer Science, COMSATS University Islamabad, Islamabad 44000, Pakistan; 2 Department of Information Technology, Bayero University Kano, Kano 700006, Nigeria; 3 Department of Electrical Engineering, Computer Engineering and Informatics, Cyprus University of Technology, Limassol 3036, Cyprus; 4 Automated Systems & Soft Computing Lab (ASSCL), Prince Sultan University, Riyadh 12435, Saudi Arabia; 5 College of Computer and Information Sciences, Prince Sultan University, Riyadh 11586, Saudi Arabia; 6 Faculty of Computers and Artificial Intelligence, Benha University, Benha 13518, Egypt; 7 Department of Computer Science and Artificial Intelligence, College of Computer Science and Engineering, University of Jeddah, Jeddah 23890, Saudi Arabia; 8 Department of ICT and Natural Sciences, Norwegian University of Science and Technology, 7034 Trondheim, Norway

**Keywords:** anomaly detection, blockchain, fraud detection, machine learning, random forest, XGboost

## Abstract

In this paper, we address the problems of fraud and anomalies in the Bitcoin network. These are common problems in e-banking and online transactions. However, as the financial sector evolves, so do the methods for fraud and anomalies. Moreover, blockchain technology is being introduced as the most secure method integrated into finance. However, along with these advanced technologies, many frauds are also increasing every year. Therefore, we propose a secure fraud detection model based on machine learning and blockchain. There are two machine learning algorithms—XGboost and random forest (RF)—used for transaction classification. The machine learning techniques train the dataset based on the fraudulent and integrated transaction patterns and predict the new incoming transactions. The blockchain technology is integrated with machine learning algorithms to detect fraudulent transactions in the Bitcoin network. In the proposed model, XGboost and random forest (RF) algorithms are used to classify transactions and predict transaction patterns. We also calculate the precision and AUC of the models to measure the accuracy. A security analysis of the proposed smart contract is also performed to show the robustness of our system. In addition, an attacker model is also proposed to protect the proposed system from attacks and vulnerabilities.

## 1. Introduction

Every industry, including banking, education, health care, and others, has modernized as a result of technological growth. Moreover, with the advent of communication technology, online transactions and means of payment are also being modernized. Through this modernization, traditional currencies are being converted into digital currencies, and all financial transactions are being conducted digitally. However, these transactions are not fully secured and are vulnerable to various digital attacks, such as fraud issues, anomalies, and privacy breaches. Additionally, as the volume of transactions rises, there is an increase in fraud associated with financial transactions. As a result, billions of dollars are lost globally every year [1]. Any suspicious activity on a network that behaves abnormally is called an anomaly. In cybersecurity and digital financial exchange, anomaly detection is used to detect fraud and network invasion. The goal of anomaly detection is to protect the network from illegal and fraudulent activities. In the financial sector, anomaly detection applications have investigated suspicious activity and identified hackers and fraudulent users. However, all anomaly detection methods in traditional financial systems are designed for centralized systems. Therefore, with the development of digital currencies, such as Bitcoin, anomaly detection methods using the blockchain are improving. Despite these advances, there are still many fraud occurrences [2]. Many artificial intelligence (AI) and machine learning techniques have been proposed to detect anomalies and fraud in digital transactions; however, there is no suitable solution for centralized systems. Blockchain is the most advanced and quickly evolving technology in many fields. It first became visible with the appearance of Bitcoin in 2008, which was introduced by Satoshi Nakamoto [3]. It addresses the security issues of centralized systems and provides solutions to external threats. It is a distributed, decentralized, and immutable ledger that time stamps all records and ensures record integrity. However, some participants in the blockchain network behave maliciously [4].

In our work, we apply existing ML techniques (i.e., XGBoost and random forest) to data in the form of blockchain transactions with the goal of detecting fraudulent transactions. To the best of our knowledge, this work is the first to investigate the application of ML to blockchain data with such an objective. The contributions of the study/work are listed below.

- Data-balancing technique and processioning are performed in the proposed system. In pre-processing, the data are divided into a training dataset and a test dataset.
- Machine learning techniques, XGboost and random forest (RF), are used for data classification. They classify the data as fraudulent or non-fraudulent. Both classifiers predict the type of data. These machine learning models are directly connected to the blockchain.
- The machine learning model is linked to the blockchain. A blockchain-based smart contract is written in which the machine learning model is deployed and used to predict the nature of new incoming transactions.
- The blockchain model is used to initiate the transactions, and then machine learning models are used to classify these transactions as malicious or legitimate.
- Two attacker models are also implemented to protect the proposed model from blockchain attacks.

The paper is organized as follows. Related work is discussed in Section 2. The proposed system model along with problem statement is presented in Section 3. Simulation results are discussed in Section 4. A security analysis is given in Section 5. Moreover, the paper’s conclusion is described in Section 6. The list of abbreviations is given in Abbreviations.

## 2. Related Work

Different public and private regions deploy blockchain technologies for various objectives because it is vital to protect and monitor auditing systems. These technologies help to evaluate its repositories and take care of the privacy of auditors. They allow auditors to send their queries in a reliable and accessible manner without exposing their identities to unauthorized users. In [5], consensus algorithms check the legitimacy of the performed transactions. However, it is inefficient to identify the transactions. Therefore, using blockchain as a solution for fraud detection does not completely address the problem. Because of this, new solutions are used to eliminate the vulnerabilities in the existing systems, such as machine learning algorithms. Different supervised machine learning techniques are used to detect fraudulent transactions. Furthermore, a comparative analysis of various machine learning methods is presented [6,7]. In [8], the authors proposed different supervised machine learning solutions to detect fake businesses. Moreover, they also tested over 300,000 accounts using random forest and XGBoost classifiers. The authors in [9] also used XGboost for accurate results. In [10], the authors dealt with the problem of an imbalanced dataset. The dataset belongs to an insurance company and describes the driving patterns of individuals. They use XGboost to predict the performance of drivers along with their telematic information.

According to [11], fraudulent activities are data mining issues because the central server for credit card transactions tells whether a trading transaction is fake or legal. Fraud detection is not a new problem; yet, there are still numerous challenges. The primary reason is that researchers lack real-time data, and banks are unwilling to share their data with researchers because customer data is confidential. At the same time, it is linked to the banks’ privacy policies [12]. In [13], the authors used a distributed data mining model to address the problems of slanted delivery of credit cards and non-uniform expenditures. A fraud detection algorithm aws presented in [14], which identifies fraud without relying on any fraudulent historical instances, with a proactive method capable of overcoming the well-known cold-start problem. In [15], The authors suggested and demonstrated the application of the uncertain association law of mining to extract useful data from credit card transactions.

The authors in [16] trainded a Support vector machine model to detect the improper data of credit card transactions. In [17], the authors mixed three different techniques to decrease the wrong beeps in fraud identification. These techniques are Bayesian learning, rule-based learning and Dempster–Shafer theory. In [18], the authors used a transaction aggregation technique to interpret the customer’s behavior before any transaction is performed and then used this aggregated data to identify fake transactions. The entire analysis takes place on the behavior of the customers. The primary purpose of the work is to develop a model that can work with unknown datasets and highlight fake datasets in them. Banks give unspecified datasets due to privacy issues. Therefore, the model behaves similarly with all the participant attributes without prioritizing them. The model has also worked on the improper datasets and arranged them in two separate sections: one for legal transactions and the other for fake transactions [18].

In [19], the authors identified the issues of trust, privacy, security and verifiability in centralized-based IoT-driven smart cities. Therefore, the authors proposed a trustworthy privacy-preserving secure framework (TP2SF) for smart cities. The proposed framework comprises three modules: a module for trustworthiness, and two modules that consist of two-layered privacy modules. The trustworthiness module is a blockchain-based reputation system that ensures the system’s security. Furthermore, two-layered privacy modules are based on an enhanced proof of work (ePoW) technique and principle component analysis (PCA). These modules transform the data into a reduced shape to prevent the system from poisoning attacks. However, a cloud system is used for data storage, which leads to a centralization problem.

In [20], the authors resolved the issue of privacy preservation through encryption techniques. They also used cryptographic approaches for the computation of data. The proposed system use asymmetric, symmetric and homomorphic encryption techniques to achieve privacy. However, high computational power and time are required to implement these approaches. Cyber attacks and intrusion detection are major problems that cause data privacy issues. Blockchain technology with deep learning algorithms is used to resolve the mentioned in [21]. These models provide security and privacy in virtual machines migrated to the cloud to protect IoT networks. The authors proposed a deep blockchain framework (DBF) model for intrusion detection based on bidirectional long short-term memory (BiLSTM) and blockchain. In [22], the authors identified the issues of centralization and cyber attacks in cloud-based systems. Therefore, they proposed a mixture-of-localization-based outliers (MLO) system with a Gaussian mixture. This collaborative anomaly detection system detects insider and outsider attacks in a cloud-based system. Privacy preservation is highly important for cyber–physical systems (CPSs). In these systems, anomaly detection systems are required to protect the system from inner and outer attacks [23]. Therefore, the authors proposed a new privacy-preserving anomaly detection framework that protects the system from attacks and keeps sensitive information confidential. The proposed method is based on two modules, i.e., the pre-processing module and anomaly detection module that used a Gaussian mixture model (GMM). However, the proposed system is inefficient for tackling modern IoT attacks.

### 2.1. Adversarial Machine Learning Methods

In adversarial machine learning, some machine learning techniques try to exploit the model’s specific vulnerabilities and take advantage of the model’s obtained information to generate some malicious attacks [24]. Some adversarial problems are discussed in the following papers. In [25], the authors gave a comprehensive overview of the research conducted in the last decade, considering the pioneering research from the security of non-deep learning algorithms to the advances in this field, i.e., properties of security in deep learning algorithms.

In [26], the authors proposed unsupervised random forest algorithms to reduce the number of fraudulent transactions. Further, this proposed algorithm was used to analyze the detection of credit card fraud. Moreover, the Bayesian network assembles a coordinated non-cyclic chart, further used for the conditional probability distribution for creating a non-cyclic graph. Results show that the random forest-based proposed algorithm performed better than its counterparts. Authors in [27] also proposed a random forest model for detailed feature selection, financial fraud detection, importance measurement of variables, and multidimensional and partial correction analysis. Nevertheless, the authors applied several statistical methodologies, i.e., non-parametric and parametric models, to detect accuracy. They concluded that non-parametric models have less accuracy compared with parametric models. In [28], the authors worked on the problem of intrusion detection in cyber security. They used a dataset which has highly sensitive training data. This type of dataset is vulnerable to cyber attacks. To resolve this issue, they used a random forest algorithm that performs better in detecting cyber attacks. However, there is still room for researchers to improve the detection of cyber attacks. In [29], the authors proposed an effective random forest classifier for anomaly detection in an IoT network. They also compared the performance of an intrusion detection system (IDS) and random forest classifier in terms of accuracy and false alarm rate. However, security is the major issue while implementing an IoT network. In [30], the authors identified the problems of malicious data and manipulation of data by an attacker. Therefore, they implemented the evasion classifier and checked its effectiveness on a test case. The authors analyzed some potential techniques used to increase the robustness of machine learning models against the attacks of data manipulation.

## 3. Problem Statement and System Model

In this section, we first explain the problem and then present our proposed system model.

### 3.1. Problem Statement

With the advancement of technology, cyber crime is also increasing day by day, and the financial sector is the most affected sector by cyber crime [5]. The main reason for this problem is security vulnerabilities in financial systems. Anomalies occur in these systems, which are also known as frauds. In traditional financial systems, credit card frauds are the most common frauds, and AI techniques are used to solve these frauds. As a result, the financial industry suffers a loss of billions of dollars each year due to these frauds [1]. In [31], the authors employed unsupervised machine learning techniques to detect the monetary anomalies. However, according to [32], supervised machine learning techniques are more effective for fraud detection. A large amount of learning data and labeled data is good for supervised learning. Therefore, the authors developed a complex model to learn the patterns of anomalies and fraud. However, this model is not able to provide accurate results. Moreover, blockchain innovation solves several fraud problems. It provides security and privacy to the financial sector, as it is decentralized and immutable. However, it does not address such issues as loss of privacy, Sybil attacks, and double-spending attacks.

The purpose of these attacks is to discourage illegal activities and increase financial benefits. Bitcoin is a digital currency based on the concept of proof of work (PoW). In the Bitcoin network, all digital transactions are executed in a distributed manner using digital signatures and hashes via a timestamp service. Bitcoin transactions do not involve a trusted third party to verify the transactions. Therefore, a user can spend the same coin twice, which becomes a fraudulent transaction and is known as a double-spending attack [33]. In [12], the authors discussed the Bitcoin theft known as “all in vain”, in which hackers stole nearly 25,000 bitcoins.

To address these issues, we propose a secure and efficient blockchain-based model with the integration of machine learning algorithms. The proposed model detects anomalies and thefts based on the predictive model. In the proposed work, machine learning models are trained on a dataset according to the fraud types and integrated transactions. The proposed model is linked with blockchain to overcome security and threats.

### 3.2. Dataset Explanation

The dataset used in this paper is downloaded from Kaggle [34]. This dataset consists of raw bitcoin transactions. These are the bitcoin transactions from the creation of bitcoin to now. This dataset contains 30 million transactions. However, due to the limited storage and computational power, only 30 thousand transactions are used. The dataset contains the 11 attributes and 30,000 observations. These attributes show the degrees of the bitcoins, mean of out and in degrees and the malicious transactions of these bitcoins. According to the dataset, there are multiple senders and receivers for a single transaction, and a single user can own multiple transaction addresses. In this network, every user is anonymous, as no relevant record is associated with the transaction address [35].

### 3.3. Proposed System Model

The proposed system model consists of two layers: blockchain and machine learning. The blockchain model initiates transactions, and then machine learning models are used to classify these transactions as malicious or legitimate. This is a binary classification. The proposed system model is based on the integration of machine learning and blockchain for fraud and anomaly detection in the financial sector. The anomaly detection system identifies unusual suspicious events that are different from most of the data. A dataset of bitcoin transactions is used for the proposed model. We also use the random forest and XGboost classifiers to classify legitimate and malicious transactions. These classifiers are also used to predict new incoming transactions. The proposed model is trained and tested for legitimate and malicious data patterns using the given dataset. The proposed system model consists of the following steps (discussed in the below subsections).

#### 3.3.1. Data Balancing Using SMOTE

Imbalance of data is a major problem in machine learning, where the distribution of classes is highly imbalanced. The accuracy of machine learning algorithms decreases due to data imbalance. It increases when the number of instances of one class is greater than the other class. Therefore, SMOTE is used to solve this problem, and synthetic samples are randomly generated for the minority class [36]. This technique solves the overfitting problem caused by random oversampling of the data. It is based on random sampling, where a data point is selected from the minority class. Then random weights are assigned to its neighbors, and these neighbors are added to the original samples. The main task of SMOTE is to synthesize the minority class samples. Data balancing improves the effectiveness of machine learning algorithms and helps to achieve better results. In Algorithm 1, SMOTE is used to balance the data, and the class distribution of the data is imbalanced. Lines 1 to 6 show input, output, and initialization of the variables. Lines 7 to 16 show the working mechanism of SMOTE for data balancing. SMOTE works on the pattern of K-nearest neighbor, where the algorithm generates synthetic data. In the first step, SMOTE selects random data from the minority class. In the second step, the K-nearest neighbors in the dataset are determined. Finally, synthetic data are generated between the randomly selected data by selecting the K-nearest neighbors. Moreover, when we train the model on the imbalance dataset, we check that the data are balanced or not if we are going to balance the data, then we first divide the data into testing and training parts and apply sampling technique only on the training data.
**Algorithm 1: **Data balancing through SMOTE1:Initialization2:**Inputs:**  Minority data M ${}^{\left(D\right)}$= $m_{i}\in$ X, Where i = 1, 2, 3, …, D3:**Outputs:**  Synthetic Data *S*4:Number of minority samples (D)5:Percentage of SMOTE (P)6:Number of (k) nearest neighbors7:**for** n = 1 to D **do**8:Find the *K* nearest neighbors of $D_{i}$9:Check $\bar{P}=P/100$10:**While**$\;\bar{P}\neq 0$**do**11:   Select a random sample *m* in minority class12:   Find neighbor of *m*13:   Pick a random number α∈[0,1]14:   $\bar{m}=m_{i}+\alpha \left(\bar{m}-m_{i}\right)$15:**While** Append $\bar{m}$ to S16:Check $\bar{P}=P-1$17: **end while**18: **end for**19:**End**

#### 3.3.2. Detection of Fraudulent Transactions

As more businesses go online, fraud and anomalies in online systems are also on the rise. Fraud detection systems that rely on static rules created by human experts have been used to combat online fraud. For this reason, organizations face a large number of fraudulent activities in online transactions that need to be minimized. In this study, we address fraudulent transactions with Bitcoins. Unusual patterns that do not conform to expected behavior, called outliers, can be detected using anomaly detection. In the proposed model, a dataset of bitcoin transactions is used. This dataset is based on bitcoin transactions in the financial sector. As we know, the transaction pattern of cryptocurrencies of bitcoins and ethers are quite similar. Therefore, we trained our model in the dataset of bitcoins, and it also gives correct prediction on the transactions of ethers. Our proposed model can work efficiently in financial sectors, where blockchain-based cryptocurrencies are used.

#### 3.3.3. XGBoost

XGboost is a boosting algorithm that generates sequential trees. There are multiple trees, and each successive tree aims to reduce the error of the previous tree and update the residual error. Therefore, each new sequential tree has the updated residual error value that is used for boosting. The proposed model uses XGboost to classify legitimate and malicious transactions. Moreover, this algorithm connects to the blockchain smart contract and predicts the new incoming transactions.

Algorithm 2 shows the working of XGboost based on the given dataset. In this algorithm, lines 1 to 3 show the inputs, outputs and the initialization of variables. Lines 4 to 8 show the testing and training of the dataset. The deployment of the model is shown in lines 9 to 11. Blockchain technology is also integrated into this algorithm from lines 12 to 17. These lines show that when a new transaction occurs in the blockchain, it passes to the XGboost to check the transaction’s integrity. The notation “if Predictions==0” in line 13 denotes that if the user passes a test sample to the trained XGboost model and it returns ‘0’ in response, then it means the specific test sample belongs to the legitimate class; otherwise, if “if Predictions==1”, then it means that it belongs to the malicious class. Furthermore, line number 12 of the algorithm explains the notation predictions. The proposed model predicts the transaction and sends it back to the blockchain with its status. In addition, the performance of the learning algorithms is improved through hyperparameter tuning. A large number of hyperparameters makes XGBoost powerful and scalable; however, it is also difficult to tune because it has a large parameter space.
**Algorithm 2: **Fraud detection through XGboost1:**Inputs:**  Balanced Dataset *S*2:**Outputs:**  Transactions in Blockchain *B*3:Initialization of Dataset4:Spliting of *S* into training and testing5:$X_{train}\leftarrow$ input variables from dataset6:$Y_{train}\leftarrow$ target variables to dataset7:$X_{test}\leftarrow$ input variables from test dataset8:$Y_{test}\leftarrow$ target variables from test dataset9:Model = XGBClassifier $\;(n_{e}stimators=100)$10:Model = Model.fit($X_{train}$, $X_{train}$)11:$Y_{pred}$ = Model.predict $\left(X_{test}\right)$12:Predictions = [round(value) for value in $Y_{pred}]$13:**if** Predictions==0 **then**14:transaction=legitimate15:*B***.add** (transaction)16:**else if** Predictions==1 **then**17:   transaction=malicious18:**end if**19:**return** *B*20:**End**

#### 3.3.4. Random Forest

Random forest is one of the most popular machine learning algorithms that is mainly used for classification. It can be used on both linear and nonlinear data. Random forest is the most productive machine learning algorithm for imbalanced datasets. A single basic classifier cannot solve the problem of an imbalanced dataset. In the proposed system, random forest is used for fraud detection in an unbalanced dataset which has a smaller number of fraud occurrences. In [37], the authors also used random forest on the imbalanced dataset. They used two types of datasets: one with the same number of fraud occurrences and one with a smaller number of fraud occurrences. However, the accuracy of the RF algorithm in the proposed model is better than the previous models. RF integrates several decision trees, where the final outcome is decided based on the majority vote. It also addresses the problem of overfitting. The training sample has a significant imbalance ratio (minority:majority = 0.001:0.999). Under these conditions, conventional classifiers may not be sufficient. In this scenario, RF is used with the benefit of keeping certain essential information about the majority class and using all available information.

### 3.4. Linkage of Blockchain with Machine Learning in the Proposed Model

Blockchain technology has been used for the past few years to provide security and privacy in various networks. Despite the fascinating features of blockchain, it is still vulnerable to fraudulent activities. The malicious entities may perform invalid and fraudulent transactions using various methods, such as a double-spending attack. In the proposed system, blockchain is combined with machine learning to solve this problem. The database of bitcoin transactions is used in the underlying work, and the proposed machine learning model is trained on the dataset. The pattern of transactions stored in the database is analyzed for further use. In parallel, the transactions are performed on the Ethereum network. The pattern of these transactions is assumed to be similar to the pattern of bitcoin transactions stored in the bitcoin transaction database. Moreover, each new Ethereum transaction is made an input to the machine learning model, and the model is trained on it. The transaction pattern is analyzed and compared with the bitcoin transaction pattern. If the pattern of both transactions matches, the new transaction is classified as legitimate or malicious. To further test the robustness of the proposed system, a double-spending attack is implemented in the underlying work.

**Figure 1:**
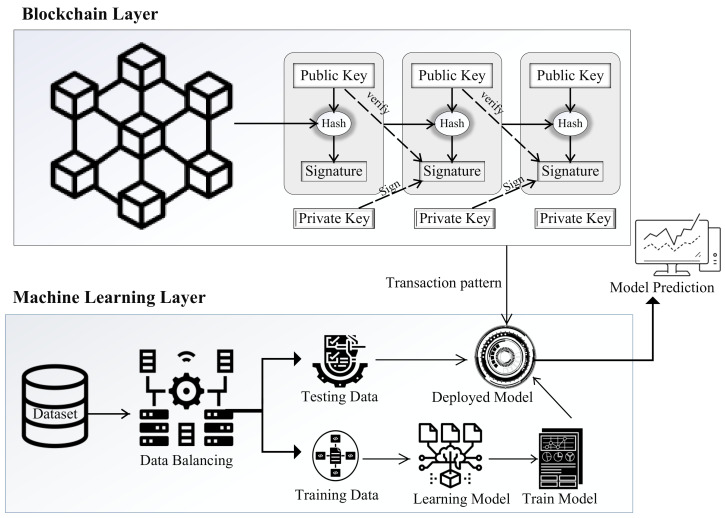
The proposed system mode of blockchain and ML.

In Figure 1, blockchain-based transactions are verified using a machine learning model, and the prediction result shows that the transaction is legitimate or malicious. The prediction of the machine learning model is based on the training and testing of a bitcoin transaction-based dataset.

## 4. Results and Discussion

This section first presents the simulation results of our proposed model, then we present the results after inducing modern cyber attacks to the system, i.e., Sybil attack, and double-spending attack.

**Figure 2:**
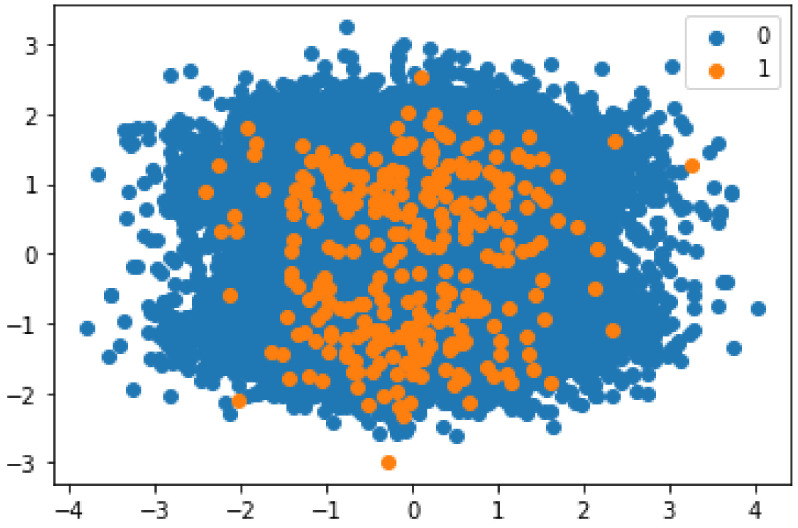
Imbalanced data.

**Figure 3:**
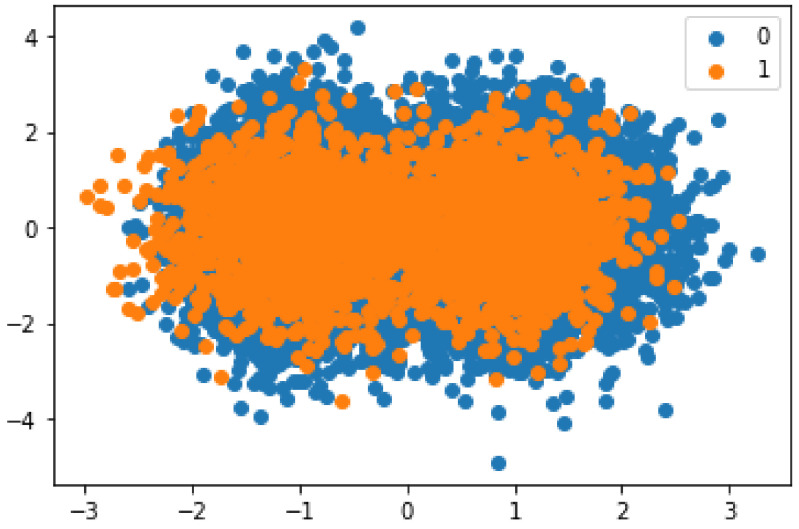
Balanced data.

The selected dataset is highly skewed, as shown in Figure 2 and Figure 3. The classification models are biased toward the majority class due to the imbalance of the data.

Figure 2 shows the presence of malicious and honest transactions in the dataset. It can be seen from the figure that the number of honest transactions is higher than the number of malicious transactions. This imbalanced nature of the data leads to a bias in the classification. Synthetic data are used to solve this problem. The malicious entities are oversampled using SMOTE. The synthesized transactions are added to the dataset to limit the bias of the model during classification. The results obtained after using SMOTE are shown in Figure 3.

**Figure 4:**
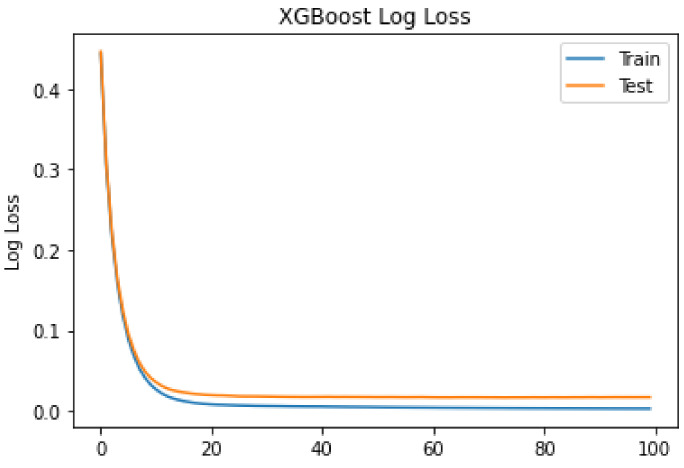
Logloss of XGboost.

The observed log loss of XGBoost during training is shown in Figure 4. The log loss is observed for both the training data and the test data. From the figure, it can be seen that at a count of 10 iterations, a drastic drop is observed for both the training and test data. Moreover, the smoothness of the curves indicates that the model efficiently captures the nonlinear patterns of the data. For the test data, the log loss is higher than for the training data. However, the difference is not too large. The smaller difference between the training and test curves indicates that the model is well trained on unseen data. The trained model can be applied to real-world scenarios for anomaly detection in blockchain networks.

**Figure 5:**
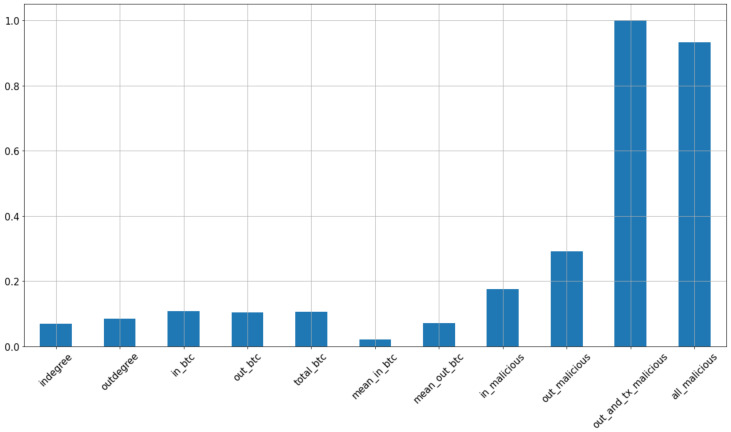
Correlation with class fraudulent or not.

Figure 5 shows the correlation between the fraudulent and non-fraudulent class. The correlation value 1 observed for $out_{a}nd_{t}x_{m}alicious$ shows the maximum correlation. Meanwhile, the value almost equal to 0, in the case of $mean_{i}n_{b}tc$, shows the minimum correlation between fraudulent and non-fraudulent.

**Figure 6:**
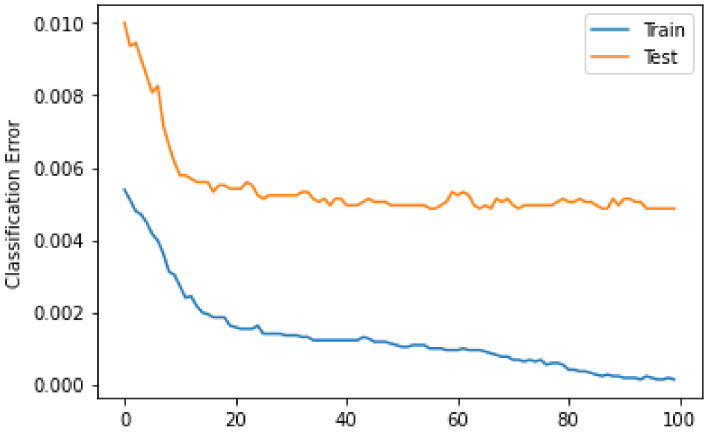
Classification error of XGboost.

Figure 6 shows the error that occurs when classifying with XGBoost. It shows the error for both training and test data. It can be observed that the classification error decreases as the number of iterations increases. The error is high for training data, and the figure shows a gradual decrease, while it is lower for test data and decreases rapidly.

**Figure 7:**
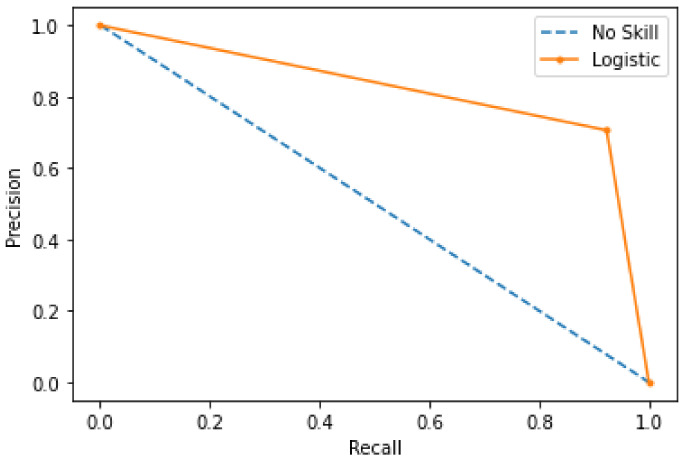
Precision of RF.

**Figure 8:**
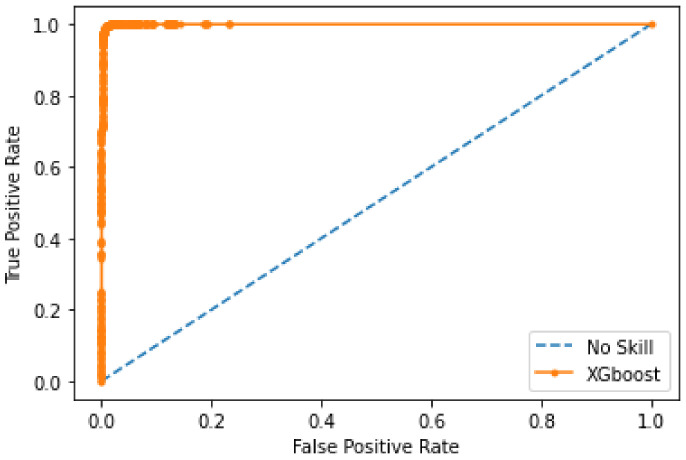
Accuracy of XGboost.

The precision–recall curve of the XGboost model is visualized in Figure 7. This curve predicts the harmonic mean of both precision and recall. It is seen that a very slight decrease is observed, starting from 1. As soon as the recall value reaches more than 0.9, there is a sudden drop in the precision value. Figure 8 shows the accuracy when XGBoost is used. It shows that the highest peak of 0 to 1 indicates that the model achieves optimal accuracy in classifying blockchain transactions as legitimate or malicious. After reaching the maximum value of 0.9, the accuracy remains constant throughout the training.

**Figure 9:**
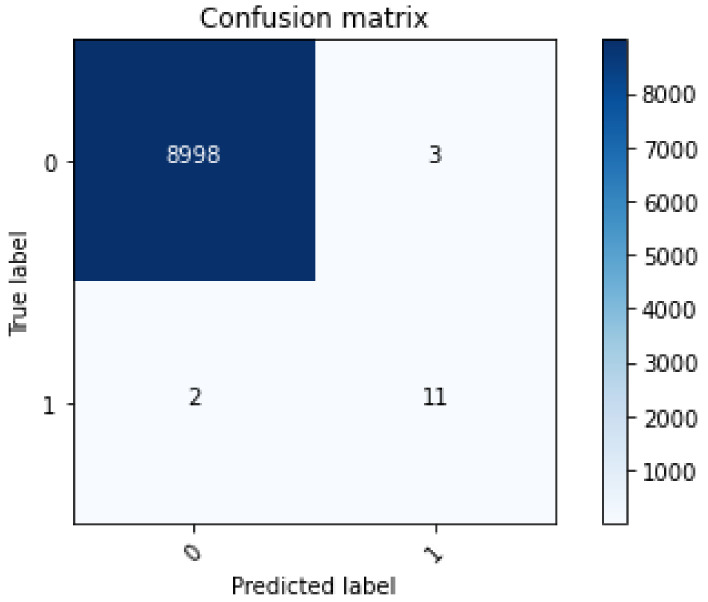
Confusion matrix through random forest.

**Figure 10:**
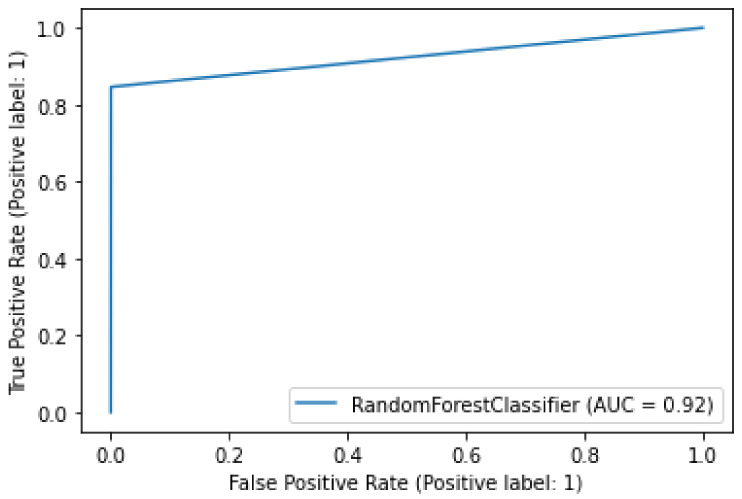
Accuracy of random forest.

Figure 9 shows the confusion matrix obtained using RF. In this matrix, random forest selects 9014 random samples, correctly identifying 9009 predictions. This means that the proposed model efficiently discriminates between malicious and legitimate transactions. The matrix shows that the highest values are obtained in the case of true negatives, namely 99%. In the other three cases, the number of values is lower. This shows that the proposed model is efficient in detecting true negative transactions. Moreover, the phenomenon of majority voting in the random forest increases the performance of the model during classification. Figure 10 shows the AUC of a random forest. The AUC describes how well the model distinguishes between the positive and negative classes. It can be seen that the value of the AUC increases dramatically at the beginning to almost 0.85. Thereafter, a gradual increase is observed until the maximum value of 0.92 AUC is reached. The random forest model achieves an AUC of 0.92, which means that it performs well in capturing legitimate and malicious transactions.

**Figure 11:**
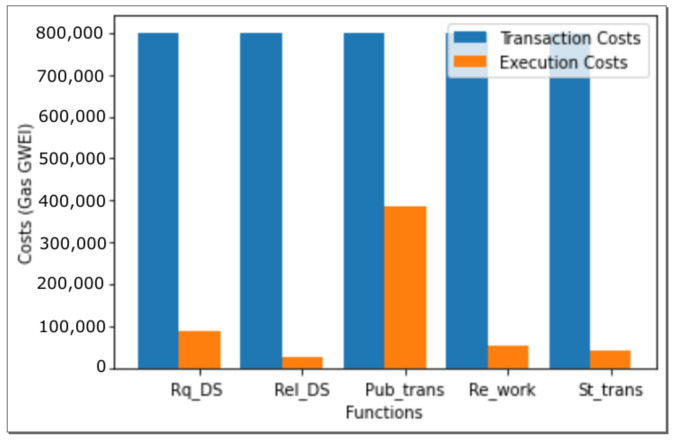
Transactions published and stored on blockchain (where Rq_DS = request dataset, Rel_DS = release dataset, Pub_trans = public transaction, Re_work = reuse work, and St_trans = store transaction).

Figure 11 shows the transaction and execution costs incurred in executing the functions involved in the blockchain smart contract. The costs are expressed in terms of gas, a basic unit of gas consumption in the blockchain network. From the figure, it can be seen that the transaction costs of all functions remain the same, while the execution costs of the publish transaction function are the highest, as mining costs are also included. Overall, the transaction costs are higher than the execution costs for all functions. The reason for this is that the former includes the processing costs of entire transactions, while the latter includes only the execution costs of some operations in a given function.

### 4.1. Validation of Proposed Model Based on Modern Cyber Attacks

Nowadays, blockchain technology is considered the most secure technology for financial transactions due to its advances; however, it is still vulnerable to current cyber attacks. Despite all the advances and security measures, some advanced cyber criminals find strong attacks against the blockchain. The security features of blockchain cannot maintain its security measures against modern cyber attacks, such as selfish mining attacks, Sybil attacks, double-spending attacks, and replay attacks [38]. Therefore, this section explicitly presents results of our proposed model when modern cyber attacks are induced in the system.

#### 4.1.1. Double-Spending Attack

In the blockchain, a transaction is only confirmed after the agreement/verification of all nodes. This verification takes a specific period, which creates a chance for cyber attacks. Double spending is one of these attacks that exploit the transaction verification time. Every transaction on the blockchain takes time for verification, and attackers use this time to their advantage. During the transaction verification delay, the attacker uses the same coin at two places as the verification of both transactions takes place simultaneously. In this way, digital currency is duplicated and falsified easily. In Ref. [33], the authors worked on the two double-spending attacker models. They enhance the two existing attacker models of Satoshi Nakamoto and Rosenfield for double spending. The first proposed model is called the “generalized model”, in which authors added a time parameter. This parameter is used to calculate the time advantage of an attacker. The second proposed model is known as the time-based model. This model counts the time when an attacker and honest node mined their last blocks.

The parameters used in both models have the same definitions and use similar notions. The parameters used in the proposed model are given Abbreviations.

The authors discussed the given equations in Ref. [33]. These equations help to evaluate the probability that a double-spending attack can occur in a blockchain network. The probability of a double-spending attack is given in terms of the attacker progressing from 1 block to *n* blocks and ending up at the difference of K−n blocks. It is given in Equation (1).

(1) $$DS_{N}\left(q,K\right)=\sum_{n=0}^{+\infty }P_{N}\left(q,K,n\right)C_{N}\left(q,K-n-1\right)=1-\sum_{n=0}^{K}P_{N}\left(q,K,n\right)\left(1-C_{N}\left(q,K-n-1\right)\right)$$

In Equation (1), $C_{N}$ is a catch-up function used to define the probability of a double-spending attack. This probability is calculated by the expected branch length of the attacker. Moreover, in the given equation, the catch-up function depends upon a random walk in which the mining reward is given to the honest or attacker node.

$$C\left(q,z\right)\left\{\begin{array}{c} {\left(\frac{q}{p}\right)}^{z+1}\;,\;if\;q<0.5\wedge z>0\\ 1\;\;\;\;,\;otherwise. \end{array}\right.$$

In the given equation, *q* defines the computational power of the attacker, and p=1−q calculates the probability that an attacker has fewer computational resources. Moreover, *z* denotes the initial disadvantage of the attacker. *K* denotes the number of confirmations to declare a block, and *n* denotes the number of blocks mined by the attacker. The probability that the attacker is successful in mining the block before the honest block is given in Equation (2).

$$P\left(T_{q}<T_{p}\right)=\int_{0}^{\infty }P\left(T_{q}=x\right)P\left(T_{p}>x\right)dx$$

$$\;\;\;\;=\int_{0}^{\infty }\frac{q}{\tau }e^{\frac{-q}{\tau }x}e^{\frac{-p}{\tau }x}dx$$

$$=q\int_{0}^{\infty }\frac{1}{\tau }e^{\frac{-1}{\tau }x}dx$$

(2) =q

where $T_{q}$ and $T_{p}$ are the random variables that are used to calculate the mining time of an honest node and an attacker node, respectively.

The attacker’s potential progress function is defined using Equation (3).

(3) $$P\left(q,m,n,t\right)=\sum_{z=0}^{n}a\left(q,t,z\right)P_{N}\left(q,m,n-z\right)$$

where

$$a\left(q,t,n\right)=\left\{\begin{array}{c} 1\;\;\;\;\;\;,\;if\;t=n=0\\ 0\;\;\;\;\;\;,\;if\;t<=0\\ \frac{{\left(qt\right)}^{n}}{n!}e^{-qt},\;,\;otherwise \end{array}\right.$$

In Equation (3), the P(q,m,n,t) is used to calculate the probability of in how much time an attacker can mine the nth block before the honest node mines the mth block. Furthermore, $P_{N}$ shows the potential progress function, and a(q,t,n) is used to calculate the probability of mining the nth block in tτ seconds.

**Figure 12:**
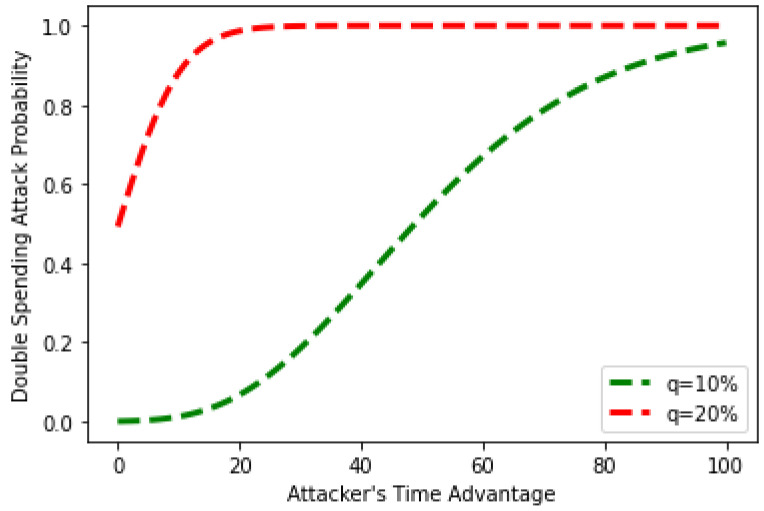
Double spending against time advantage of the attacker.

In the proposed work, the impact of a double-spending attack is assessed using the time advantage, computational power, and the number of pre-mined blocks. The number of pre-mined blocks is utilized as an input in Figure 12. The double-spending attack occurs after only a few blocks are created for values of *q* greater than 40%. It means that as the value of *q* rises, the probability rises with it, and once an attacker has control over the network, the chances of a double-spending attack become high. The probabilistic values close to 0 indicate that the double-spending attack will fail, while values close to 1 indicate a more significant success percentage for the double-spending attack.

#### 4.1.2. Sybil Attack

Blockchain has become the most secure platform for digital currency transactions. However, it is vulnerable to blockchain-based attacks, such as the Sybil attack. In a Sybil attack, a user creates multiple identities (IDs) to receive more rewards from the network or to rate himself highly. In the network, some malicious users are present and act maliciously at some point. Fake IDs are used by malicious users to obtain high ratings and deceive the network’s legitimate users. It also manipulates the network and its data. All users in the proposed system are registered and have an account. When a registered user engages in bad behavior, several false IDs that are not registered on the network are created. In [39], the authors proposed an equation related to the probability of a Sybil attack, which is given below:

(4) P(w)=nswN−1N−wN+ns−1N

In the given equation, *N* represents the number of honest nodes’ identities, and ns represents the successful Sybil node’s identities. Suppose at the initial stage, *w* is the total identities in the network, which is calculated by using this w=N+ns−1. The probability of an attack is increased when the number of successful Sybil identities is increased in the network. On the other hand, the attacker fails to implement the Sybil attack if the Sybil identities are less than the honest identities. The mentioned equations are hypergeometric equations.

**Figure 13:**
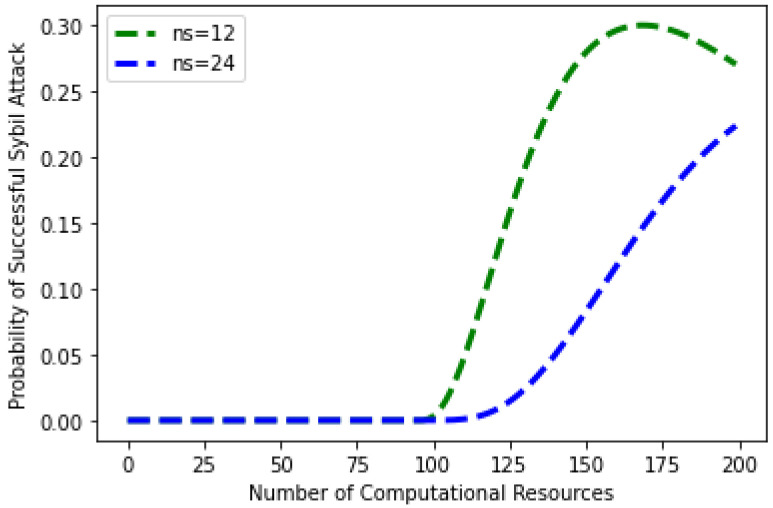
Probability of Sybil attack versus number of Sybil identities.

In Figure 13, the evaluating parameters of Sybil attack are given, such as different Sybil identities ns = 12 and 24, number of nodes, and the computational power of the attacker node. The given figure shows the probability and impact of different Sybil identities in the network. It is observed from the figure that when the number of Sybil identities is 12, and computational resources are 0, then the probability of a Sybil attack is zero. However, the probability of a Sybil attack is increased when the computational resources are increased from 100 with 12 Sybil identities. It shows that if the attacker increases the computational resources, the probability of a Sybil attack becomes high. Moreover, when the Sybil identities are increased up to 24 with the computation resources equal to 125, the probability of a Sybil attack is zero. However, when the computational resources of Sybil identities are increased beyond 125, the probability of an attack is also increased. The graph depicts that the probability of a Sybil attack becomes high when the number of Sybil identities and computational resources is high. The findings reveal that the number of Sybil identities established by hostile people determines the likelihood of a Sybil assault. The mathematical definition of the probability of a Sybil attack’s success is shown in Equation (4).

The idea of a Sybil attack was proposed in [39] to prevent the networks from this attack. The chance of a Sybil assault is calculated in this attacker model, utilizing several characteristics, such as computational power, the number of honest nodes, and the number of fake IDs. When both the number of fake IDs and computational power increase, the likelihood of the Sybil attack increases. In a Sybil attack, the following parameters are employed.

(5) P(w)=ghQ−1N∗−hg+h−1h∗

- *Q*: number of population
- *g*: number of items in the population that are classified as success
- *h*: number of items in the sample that are classified as successes
- *c*: number of computational power of sample
- N*: number of items in the sample

**Figure 14:**
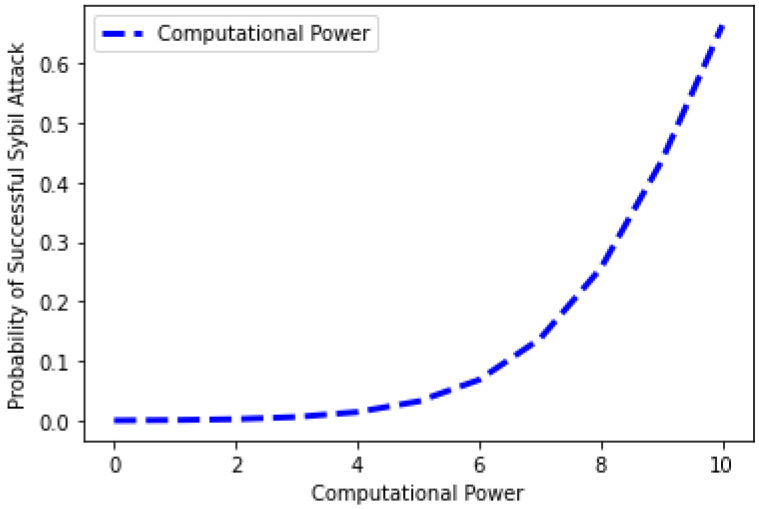
Sybil attack against computing power.

The relationship between the attack’s probability and processing power is depicted in Figure 14. The graphical representation shows that the probability of an attack increases as the computational power employed by malevolent users and fake IDs increases. When malicious users use less processing power, the likelihood of an attack decreases, and vice versa. Equation (5) gives the mathematical description of the chance of a Sybil assault succeeding against computational power.

## 5. Security Analysis

In this section, we analyze the vulnerabilities of the proposed smart contracts. The security analysis of the proposed system is discussed in detail. For the security analysis, we used Oyente software, an open-source tool developed by the authors of [40]. It analyzes the smart contract using symbolic execution techniques based upon the execution of step-wise functions [41]. Oyente software provides a flexible environment, which directly works with the Ethereum Virtual Machine (EVM) and does not require access to high-level representations, such as Solidity and Serpent [42]. Moreover, it is also used to analyze smart contracts against the following significant vulnerabilities:

- Re-entrancy vulnerability;
- Timestamp dependency;
- Callstack depth vulnerability;
- Transaction ordering dependency;
- Parity multisig bug;
- Integer overflow;
- Integer underflow.

**Figure 15:**
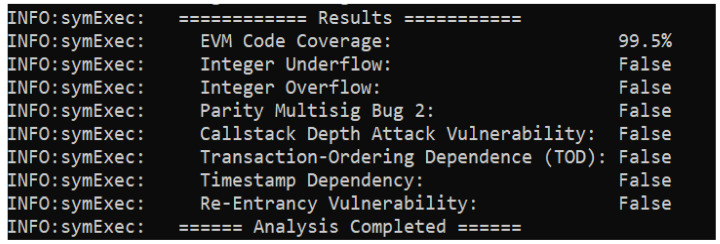
Security analysis of the proposed smart contract.

Figure 15 shows the security analysis of the smart contract involved in the proposed model. From the figure, it is observed that the outputs of all results in the analysis report are “False”, which means that the smart contract used in the proposed system model is robust against well-known vulnerabilities. All of the results being false means the proposed model is secure and robust against these attacks.

### Security Features

In this section, we discussed the solutions of our security model, and how it deals with the security threats and ensures the security of the system. The proposed solution consists of blockchain features. These features are decentralization, integrity, non-repudiation, availability and trust. This system is protected against replay attacks and man-in-the-middle (MITM) attacks.

**Integrity:** is an important feature which is used to ensure that there is no occurrence of data modification. The immutability of blockchain ensures data integrity and exchange messages between all participants and generates logs and events.
**Availability:** it makes sure that the deployed smart contract in the blockchain is always available for all participants. Availability also ensures that all services are always available. It also protects the system against denial of service (DoS) attacks because all transactions are stored in a distributed ledger of Ethereum. Therefore, there is no fear of hacking, failure and compromise. The ledger of Ethereum is highly robust against the DoS attack because thousands of trusted mining nodes protect this ledger.
**Confidentiality:** the requirement of confidentiality is achieved using a permissioned or private blockchain, e.g., Hyperledger or private Ethereum networks. The proposed system is based on a permissioned blockchain network in the proposed scenario.

## 6. Conclusions

Nowadays, blockchain is the latest and most secure technology that covers various research areas related to security. Blockchain development is based on digital currencies and is used to secure digital financial transactions. It protects financial systems from fraudulent attacks. Therefore, a blockchain-based machine learning algorithm is proposed to secure digital transactions. The proposed model predicts whether the incoming transaction in the blockchain is fraudulent or not. The proposed machine learning algorithms are trained and tested on a bitcoin-based dataset based on bitcoin transactions and predict the behavior of the incoming transactions. The given dataset is based on 30,047 entities, with smaller numbers of fraudulent entities. Due to the small amount of fraudulent data in the dataset, good results cannot be obtained because of the data imbalance problem. Therefore, we generate synthetic malicious data points through SMOTE to achieve better results. We use XGboost and random forest to classify the model and calculate the confusion matrix. This classification allows the model to distinguish between fraudulent and real data. The simulation results show that the proposed algorithm works adequately to find transaction fraud. Moreover, two attacker models are implemented to check the efficacy of the system against bugs and attacks. The proposed system is robust against double-spending and Sybil attacks.

A major limitation of our proposal is that it can be affected by the adversarial attack described in Section 2.1; we leave it to future work to address such a threat. 

## Data Availability

Not applicable.

## Abbreviations

List of abbreviations:	
**Abbreviation**	**Full Form**
AI	Artificial Intelligent
ANN	Artificial Neural Network
CPS	Cyber–Physical System
DBF	Deep Blockchain Framework
DTR	Decision Tree Regression
ePoW	enhanced Proof of Work
GMM	Gaussian Mixture Model
IoT	Internet of Things
LSTM	Long Short-Term Memory
MLO	Mixture Localization-based Outliers
MLP	Multi Layer Perceptron
PCA	Principle Component Analysis
RFE	Recursive Feature Elimination
SDA	Stacked De-noising Autoencoders
SMOTE	Synthetic Minority Oversampling Technique
SVD	Singular Value Decomposition
SVM	Support Vector Machine
XGboost	eXtreme Gradient Boosting
List of acronyms:
**Abbreviation**	**Full Form**
$C_{N}$	Catch-up function
*K*	Number of confirmation to declare a block
*m*	Honest nodes mine the block
*n*	Attackers mine the block
$P_{N}$	Potential progress function
*q*	Probability of attack
*T*	Time needed for mining
*t*	Time advantage for the attackers
τ	Average time for the mining of block
*x*	Available computational power in network
*z*	Initial disadvantage of attacker
